# Semen proteome and transcriptome of the endangered black-footed ferret (*Mustela nigripes*) show association with the environment and fertility outcome

**DOI:** 10.1038/s41598-024-57096-w

**Published:** 2024-03-25

**Authors:** Nadya Ali, Olga Amelkina, Rachel M. Santymire, Klaus-Peter Koepfli, Pierre Comizzoli, Juan M. Vazquez

**Affiliations:** 1 Committee on Evolutionary Biology, University of Chicago, Chicago, IL USA; 2 Smithsonian’s National Zoo and Conservation Biology Institute, Washington D.C., USA; 3 Biology Department, Georgia State University, Atlanta, GA USA; 4 Smithsonian-Mason School of Conservation, George Mason University, Front Royal, VA USA; 5 Department of Integrative Biology, University of California, Berkeley, USA

**Keywords:** Computational biology and bioinformatics, Genetics, Reproductive biology

## Abstract

The ex situ population of the endangered black-footed ferret (*Mustela nigripes*) has been experiencing declines in reproductive success over the past 30 years of human-managed care. A potential cause may be environmental-dependent inbreeding depression with diet being one of the contributing factors since ferrets are not fed their natural diet of prairie dogs. Here, we generated and analyzed semen proteome and transcriptome data from both wild and ex situ ferrets maintained on various diets. We identified 1757 proteins across all samples, with 149 proteins unique to the semen of wild ferrets and forming a ribosomal predicted protein–protein interaction cluster. Wild ferrets also differed from ex situ ferrets in their transcriptomic profile, showing enrichment in ribosomal RNA processing and potassium ion transport. Successful fertility outcomes documented for ex situ ferrets showed the strongest association with the semen transcriptome, with enrichment in genes involved in translation initiation and focal adhesion. Fertility also synergized with the effect of diet on differentially expressed transcriptomes, mainly affecting genes enriched in mitochondrial function. Our data and functional networks are important for understanding the causes and mechanisms of declining fertility in the ex situ ferret population and can be used as a resource for future conservation efforts.

## Introduction

As one of the world's most endangered mammals, endemic to one of the world's most endangered ecosystems, the black-footed ferret (hereafter, ferret; *Mustela nigripes*) is a textbook case for anthropogenic-caused population decline^1,2^. Although ex situ breeding successfully rescued the ferret from extinction, inbreeding depression continues to impact their reproductive success and chances for recovery^1,3^. In captivity, pregnancy success rates dropped from 60% in the 1990s to 46% in 2021^4^, concurrent with a steady decline in normal spermatozoa (hereafter, sperm) morphology in males, which has fallen from 50% in 1990 to 35% in 2021^3^ (R.M. Santymire, Pers. comm.). While previous research has demonstrated female traits play a limited effect on pregnancy and litter size, studies have determined that the percent of morphologically normal sperm in ejaculate is highly correlated with fertility^3,5–7^, and the decline in ferret whelping rates correlates with this decline in normal sperm^3^. However, wild ferrets, who are descendants of the ex situ ferrets, have improved reproductive health (T.M. Livieri, Pers. comm.), with 57.5% morphologically normal sperm in 2020 (R.M. Santymire, Pers. comm). Unfortunately, it is difficult to assess wild female reproductive success due to kits staying in burrows until nearly adult size and the restrictions to conduct research during this critical time. However, signs of past lactation are evident of litter production and most wild females do show signs of lactation (T.M. Livieri, Pers. comm.). The improved reproductive health in wild ferrets compared to ex situ populations is suggestive of the presence of environmental-dependent inbreeding depression^8^, the theory of which posits that only under certain environments will deleterious mutations cause a serious impact on fitness. In the case of the ferret, deleterious mutations caused by inbreeding depression may not negatively impact wild individuals to the same extent as ex situ individuals because the conditions encountered by the former group do not exacerbate them, as evidenced by their higher reproductive fitness (T. M. Livieri, Pers, comm).

One major difference between wild and ex situ ferrets is their diet. For tens of thousands of years black-footed ferrets evolved to be obligate predators of prairie dogs (genus *Cynomys*) on the North American plains and, therefore, obtain their balanced metabolic and nutritional needs primarily from this source of food^1^. On the other hand, ex situ ferrets are fed a standardized, commercially-produced small carnivore diet (TOR; Milliken Meats Products) along with rat, mouse or hamster carcasses for supplementation^9^. A decline in captive male ejaculate health began the year after it was decided that individual ex situ breeding facilities needed to standardize ferret diets across the Species Survival Plan® (managed through the Association of Zoos and Aquarium or AZA). The new standardized TOR diet consists of horse meat and could potentially induce oxidative-stress in sperm cells^10,11^ by being high in vitamin A, which can overwhelm the antioxidant defense mechanisms in a cell system^12^, as well as high in polyunsaturated fatty acids making sperm more susceptible to oxidative stress damage^13^. This can impact sperm morphology, sperm viability, sperm motility, DNA integrity and thereby fertility^14^. Moreover, diet not only impacts cells morphologically but has been shown to have cascading impacts on physiological and biochemical adaptations caused by transcriptomic changes^15^. Notably, diet has been shown to exert influence on gene expression in gametes, thereby impacting phenotypes^16,17^.

In our current study, we aimed to characterize the semen proteome and transcriptome of wild and ex situ ferrets on different diets. Various studies have demonstrated an association between fertility and semen protein/RNA content^18–20^, and, therefore, we also aimed to investigate this potential association in ferrets. We took advantage of an existing diet study that ex situ ferrets were being subjected to and collected samples for proteomic and transcriptomic analyses. Ferrets were part of one of three diet treatments: the control diet (TOR + two prey items per week), the control diet supplemented with vitamin E, and a prey-only diet that simulates a wild diet, over multiple generations. It was previously found that captive-born, reintroduced males did not have improved semen quality after living in the wild for almost two years (R.M. Santymire, Pers. comm.). Even though reintroduced males did not have improved semen quality, a 2015 diet study found that supplementing carcass to the control diet improved ejaculate characteristics of captive males^10^. When only supplemented with vitamin E and not with carcasses, ferrets did not have improved sperm parameters. Additionally, it was determined that adding carcass to the ferret diet reduced fecal glucocorticoid metabolites, which were used as an indication of stress, and when ferrets were fed vitamin E, fecal androgen metabolites increased during the breeding season^11^. Because of these results, adding whole carcass to the diet twice a week to the control diet is now the standard feeding protocol. The limitation of the 2015 study was that semen was only evaluated during one breeding season. We now theorize that vitamin E may have a generational effect because even though the captive-born reintroduced males did not have improved semen quality, their wild-born sons did have 50% normal sperm morphology (R.M. Santymire, Pers. comm.). Numerous studies have found that supplementing vitamin E to the diet can improve semen quality, since antioxidants neutralize attack by reactive oxygen species^21,22^.

We hypothesize that the observed semen quality and fertility status in black-footed ferrets may be linked to environmental effects of diet on semen proteome and transcriptome. To investigate this potential link, we generated and analyzed proteomic and transcriptomic data from ejaculates of both wild and ex situ ferrets utilizing the recently generated black-footed ferret genome assembly^23^. By establishing the baseline of gene expression in ferret semen, we can explore how ex situ ferrets on different diets diverge, providing insight into which diet most reflects the wild expression patterns, particularly in key fertility genes.

## Results

### Proteome of the black-footed ferret ejaculate

#### Overview of the acquired proteome dataset

**Figure 1:**
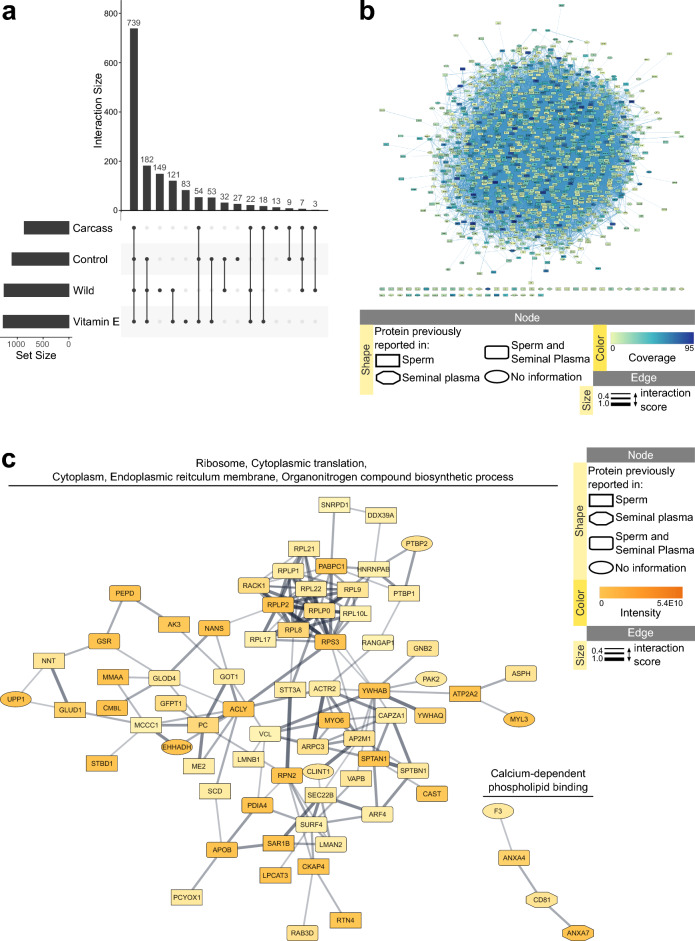
Semen proteome of black-footed ferrets. (**a**) UpSet plot showing distribution of proteins with detected signal intensity throughout diet groups. (**b**) STRING networks of predicted protein interactions for all detected proteins and (**c**) for proteins with detected intensity only in the Wild diet group. Protein interaction clusters were analyzed for functional enrichment with StringApp; clusters with no enrichment were removed from visualization. Supplementary Archive 1 contains the web session of the STRING networks for interactive viewing.

Males from five diet groups (Table 1) were used for proteomic profiling and samples were pooled for Wild (n = 4), Control (n = 9), Carcass (n = 2), Vitamin E 2nd generation (VitE2, n = 7), and mix of Vitamin E 1^st^ generation (VitE1) and VitE2 (VitE.mix, n = 3, Supplementary Table S1.1). High-resolution mass spectrometry analysis resulted in the identification of 1757 proteins (Supplementary Table S1.2). Due to peak quality, some of the peptides could not be quantified, resulting in 245 proteins with 0 intensity in all samples (Supplementary Table S1.2). The remaining proteins with calculated intensity had different distributions across the five diet groups, with 739 proteins expressed in all analyzed samples (Fig. 1a).

**Table 1:** Information on the black-footed ferrets used in this study and in the corresponding analyses.

Study ID	Animal ID (studbook number)	Name	Source location	Age at collection	Diet	Sperm conc, 10^6^/ml	Used for analysis
Car1	9469	Doublemint	FCC	1y10m	Carcass	166.3	Proteome, Transcriptome, DE
Car2	9452	Rockbiter	FCC	1y10m	Carcass	407.8	Proteome, Transcriptome, DE
C01	9571	Arthur	FCC	1y9m	Control	151.8	Transcriptome, DE
C02	9592	Calo	FCC	1y9m	Control	236	Proteome, Transcriptome, DE
C03	9519	Bannner	FCC	1y9m	Control	225.3	Proteome, Transcriptome, DE
C04	9430	Cushman	FCC	1y10m	Control	472.2	Proteome, Transcriptome, DE
C05	9350	Boatman	LZG	1y11m	Control	32.8*	Proteome, Transcriptome
C06	9128	Hawksbill	FCC	2y10m	Control	239.3	Proteome, Transcriptome, DE
C07	9276	Wilde	LZG	2y10m	Control	240.1	Proteome, Transcriptome, DE
C08	9291	Kibosh	FCC	2y9m	Control	163.6	Proteome, Transcriptome, DE
C09	9443	Meusli	LZG	1y10m	Control	63.3	Transcriptome
C10	9576	Pikachu	FCC	1y9m	Control	409.9	Proteome, Transcriptome, DE
C11	9802	Ferretson Ford	FCC	0y9m	Control	196.7	Proteome
Var1	9051	Tater	FCC	3y9m	Various	195.8	Transcriptome, DE
Var2	9072	Woodford	FCC	3y9m	Various	256	Transcriptome, DE
VE1.1	8995	Captain Barnacles	FCC	3y10m	VitE1	919.4	Proteome, Transcriptome, DE
VE1.2	8593	Dash	FCC	4y11m	VitE1	343.9	Proteome, Transcriptome, DE
VE2.01	9227	Balthasar	FCC	2y10m	VitE2	489.3	Proteome, Transcriptome, DE
VE2.02	9247	Bosler	FCC	2y10m	VitE2	269.7	Transcriptome, DE
VE2.03	9219	Cameron	FCC	2y10m	VitE2	255.2	Transcriptome, DE
VE2.04	9476	Flatback	FCC	1y10m	VitE2	237.9	Transcriptome, DE
VE2.05	9311	HopperStoppeer	FCC	2y9m	VitE2	535.9	Proteome, Transcriptome, DE
VE2.06	9135	Heavenly	FCC	2y10m	VitE2	723.4	Proteome, Transcriptome, DE
VE2.07	9150	Will Ferret	FCC	2y10m	VitE2	116.5	Transcriptome, DE
VE2.08	9181	Oslo	FCC	2y10m	VitE2	438.3	Proteome, Transcriptome, DE
VE2.09	9194	Statler	FCC	2y10m	VitE2	428	Proteome, Transcriptome, DE
VE2.10	9195	Waldorf	FCC	2y10m	VitE2	77.9	Proteome, Transcriptome
VE2.11	9318	Myrcenary	FCC	2y9m	VitE2	303.3	Proteome, Transcriptome, DE
VE2.12	9373	Martini	FCC	1y11m	VitE2	231.8	Proteome, Transcriptome, DE
VE2.13	9568	Wellington	FCC	1y9m	VitE2	228.3	Transcriptome, DE
W1	13	RMA13	RMA	NA	Wild	648.9	Proteome, Transcriptome, DE
W2	14	RMA14	RMA	NA	Wild	323.8	Proteome, Transcriptome, DE
W3	15	RMA15	RMA	NA	Wild	337.9	Proteome, Transcriptome, DE
W4	16	RMA16	RMA	NA	Wild	589.6	Proteome, Transcriptome, DE

DE, Differential gene expression analysis; FCC, USFWS National Black-Footed Ferret Conservation Center, Carr, CO; LZG, Louisville Zoological Gardens, Louisville, KY); RMA, Rocky Mountain Arsenal, Colorado; VitE1/2, Vitamin E 1st/2nd generation.

*sample had urine contamination.

#### Overlap with proteins previously reported in human semen

All proteins were checked against the protein list reported in human sperm^24,25^ and seminal plasma^26,27^, resulting in 1281 proteins being previously reported in sperm and 612 in seminal plasma, with 590 reported in both. Additionally, reported localization in the sperm was also checked^25^, resulting in 1228 proteins previously reported in whole sperm, 86 in the sperm head and 639 in the sperm tail. Finally, genital tract protein markers previously reported in seminal plasma were checked^26^, resulting in identification of 29 testis, 14 epididymis, 2 seminal vesicle, and 3 prostate markers. Supplementary Table S1.2 presents the information of all identified proteins with corresponding information from previous reports.

#### Predicted posttranslational modifications

Four different posttranslational modifications (PTMs) were analyzed, including phosphorylation, carbamidomethylation, acetylation and oxidation (Supplementary Table S1.2, Supplementary Table S2). Carbamidomethylation was the most common PTM predicted in 842 proteins, followed by 511 proteins with oxidation, 157 with acetylation, and 82 with phosphorylation. A-kinase anchor protein 4 (AKAP4) showed all four PTMs with the highest number of observations (Supplementary Table S1.2).

#### Majority of proteins demonstrated predicted interactions between each other with enrichment in various functions and pathways

The full list of proteins (1757) was subjected to predicted protein–protein interaction analysis using the StringApp in Cytoscape and resulted in one big “hairball” with only 54 proteins not connecting (minimum interaction score used was 0.4, Fig. 1b). Adding a clustering step using the Markov Cluster Algorithm (MCL) resulted in unraveling the hairball into many clusters that could then be grouped based on the enrichment analysis of each of them. The largest cluster was enriched in mitochondrion function, including oxidative phosphorylation and glycolysis; it was grouped with the clusters related to metabolism (Table 2, Supplementary Fig. S1). The next largest cluster was enriched in proteasome and chaperon complexes and grouped with clusters related to protein folding, processing, and transport (Table 2, Supplementary Fig. S1). A number of clusters were enriched in sperm motility, capacitation, and actin cytoskeleton, as well as sperm-egg recognition and fusion (Table 2, Supplementary Fig. S2). A smaller number of clusters were enriched in RNA splicing and translation, with the translation cluster being one of the densest (Table 2, Supplementary Fig. S2). Finally, a small number of clusters were enriched in functions and pathways associated with extracellular matrix, including cell adhesion, coagulation, extracellular transport, and defense response to virus (Table 2, Supplementary Fig. S3). Supplementary Archive S1 contains all networks and data that can be viewed in a browser interactively (use “readme.txt” file for simple instructions). In addition to the style presented in the figures (mapped coverage, previously reported source), additional data was mapped to networks in the interactive version, including per diet expression and predicted PTMs.

**Table 2:** Representative functional terms enriched in the largest predicted protein–protein interaction clusters of semen proteome.

Nodes	Edges	Functional term	Term name	Enrichment FDR	#proteins	Network view
166	2999	Mitochondrion	GO:0005739	6.94E-126	139	Supplementary Fig. S1
		Oxidative phosphorylation	map00190	4.22E-50	43	
		Glycolysis / Gluconeogenesis	map00010	5.8E-4	7	
39	290	Carbon metabolism	map01200	8.57E-34	23	
20	125	Purine metabolism	map00230	7.4E-29	17	
		Pyrimidine metabolism	map00240	1.9E-17	10	
19	45	Glutathione metabolism	map00480	3.99E-10	7	
74	1378	Proteasome complex	GO:0000502	8.1E-61	35	Supplementary Fig. S1
		Chaperone complex	GO:0101031	7.11E-13	9	
54	345	Protein transport	GO:0015031	8.71E-34	40	
		GTPase activity	GO:0015031	8.71E-34	21	
43	219	Protein folding	GO:0006457	2.02E-13	13	
34	159	Protein processing in ER	map04141	1.77E-27	21	
46	289	Axonemal dynein complex	GO:0005858	2.03E-22	12	Supplementary Fig. S2
		Cilium-dependent cell motility	GO:0060285	1.65E-15	12	
38	160	Regulation of actin cytoskeleton	map04810	9.31E-14	13	
14	53	Actin cytoskeleton organization	GO:0030036	2.59E-14	12	
19	47	Sperm flagellum	GO:0036126	8.67E-7	6	
15	40	Motile cilium	GO:0031514	2.0E-8	7	
15	35	Single fertilization	GO:0007338	3.34E-11	8	
		Fusion of sperm to egg	GO:0007342	1.4E-4	3	
38	477	Translation	GO:0006412	2.24E-37	30	Supplementary Fig. S2
9	27	RNA splicing	GO:0008380	1.58E-09	8	
12	29	Nucleosome	GO:0000786	3.72E-7	5	
49	199	Extracellular space	GO:0005615	1.0E-22	31	Supplementary Fig. S3
		Hemostasis	GO:0007599	7.62E-6	7	
17	57	Desmosome	GO:0030057	3.46E-16	8	
		Cell–cell adhesion	GO:0098609	7.58E-5	7	
12	35	Clathrin coat	GO:0030118	5.53E-8	5	
8	11	Defense response to virus	GO:0051607	0.0031	8	

Nodes—number of proteins in the cluster; Edges—number of predicted protein–protein interactions in the cluster; #proteins—number of proteins from the cluster enriched in the term.

#### Proteome of wild ferrets contains the most unique proteins

As seen in Fig. 1a, the Wild diet group had the highest number (149) of unique proteins across all diets. When these proteins were subjected to predicted protein–protein interaction analysis via StringApp, they formed a cluster enriched in ribosomes and cytoplasmic translation (Fig. 1c, Supplementary Archive S1). Unique proteins from other diets were also checked in StringApp, but either returned no clustering (Control, Carcass) or clustering with only general enrichment in cytoplasm (VitE.mix, Supplementary Archive S1).

### Transcriptome of the ejaculate from wild and ex situ black-footed ferrets

#### Overview of the acquired transcriptome dataset

Males from all diet groups (Table 1) were used for transcriptome analysis, with one library constructed per each animal: Wild (n = 4), Control (n = 10), Carcass (n = 2), VitE1 (n = 2), VitE2 (n = 13), and Various (n = 2). The average number of genes with transcripts per million (TPM) above zero was 6502 per sample (S.D. = 2399). Supplementary Table S3.1 provides information on sequence data statistics. The acquired sequence data in fastq format was deposited into the NCBI Sequence Read Archive under BioProject accession number PRJNA997940.

#### No clear association of semen transcriptome with sperm characteristics

**Figure 2:**
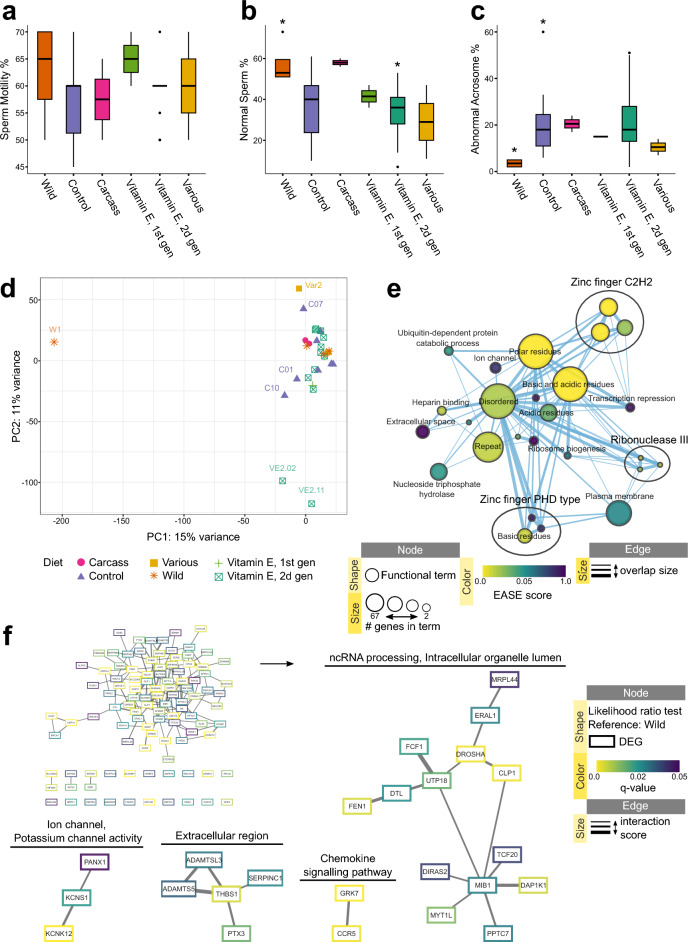
Sperm characteristics and semen transcriptomics of black-footed ferrets. (**a–c**) Box plots visualize sperm characteristics for each diet group. *groups are different with adjusted *p*-value < 0.05. (**d**) Principal component analysis plot representing variation in semen transcriptome of all groups. (**e**) EnrichmentMap displaying enriched gene-sets and (**f**) STRING network of predicted protein interactions based on differentially expressed genes between the full (all diet groups) and reduced (Wild excluded) models (Likelihood-ratio test, q-value < 0.05). STRING networks were built using either 0.1 (big cluster) or 0.4 (smaller clusters) as the starting interaction score. Protein interaction clusters were analyzed for functional enrichment with StringApp; clusters with no enrichment were removed from visualization. Supplementary Archive 2 contains the web session of both EnrichmentMap and STRING networks for interactive viewing.

Obtained data on sperm motility, normal morphology and acrosome condition was used to compare the diet groups. No significant changes were observed in sperm motility across diets (Fig. 2a); however, the percentage of sperm with normal morphology was higher in Wild compared to VitE2 (adjusted *p*-value = 0.046, Fig. 2b), while the percentages of sperm with abnormal acrosomes was lower in Wild compared to Control (adjusted *p*-value = 0.034, Fig. 2c). When data was visualized using principal component analysis, no clear association with sperm characteristics or diet could be observed (Fig. 2d); principal components 3 and 4 also did not show any distinct clustering between sample groups (Supplementary Fig. S4).

#### Wild environment conditions contribute to transcriptomic differences between groups

A likelihood-ratio test was used to compare the full model (all diets) with a reduced one (Wild reference) and resulted in 143 differentially expressed genes (Supplementary Table S3.2). These genes were mainly enriched in terms related to zinc finger and DNA binding, rRNA processing, extracellular matrix and cell adhesion, and potassium ion transport (Fig. 2e, Supplementary Fig. S5a). Predicted protein interaction analysis resulted in clustering of the genes together when setting the interaction score to a minimum of 0.1, and in a few smaller clusters when the score was set to a minimum of 0.4 (Fig. 2f). The smaller clusters returned enrichments associated with intracellular organelle, rRNA processing, potassium voltage-gated channel activity (including genes *KCNK12*, *KCNS1* and *KCNB2)* and extracellular region (Fig. 2f, Supplementary Fig. S5b). Supplementary Archive S2 provides an interactive view of the networks and data tables.

Overall, the semen transcriptome of Wild ferrets differs from the ex situ population in terms related to rRNA processing, potassium channel activity and extracellular matrix.

### Association of semen transcriptome with fertility outcome in ex situ ferrets

#### Fertility outcome has the strongest association with semen transcriptome across all factors

**Figure 3:**
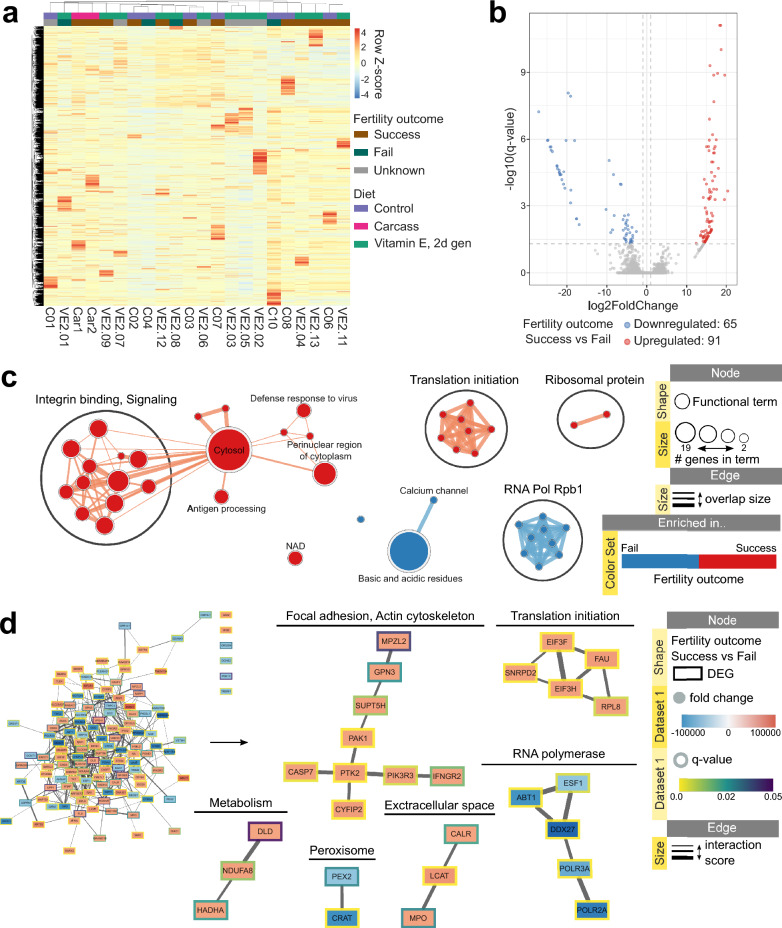
Association of semen transcriptome with fertility outcome in black-footed ferrets. (**a**) Heatmap of one-way hierarchical clustering analysis (Euclidian method, complete linkage) using Z-score for RLE normalized values of all genes expressed in semen of ferrets associated with various fertility outcomes and diet. (**b**) Volcano plot showing up-regulated and down-regulated genes, (**c**) EnrichmentMap displaying enriched gene-sets and (**d**) STRING network of predicted protein interactions, all based on differentially expressed genes in semen of ferrets with success versus fail fertility outcomes (Multi-factor design with Fertility and Diet, Wald test, q-value < 0.05, absolute fold change ≥ 2). STRING networks were built using either 0.1 (big cluster) or 0.4 (smaller clusters) as the starting interaction score. Protein interaction clusters were analyzed for functional enrichment with StringApp; clusters with no enrichment were removed from visualization. Supplementary Archive 2 contains the web session of both the EnrichmentMap and STRING networks for interactive viewing.

Information on fertility outcome was available for some ferrets from three different diets: Control (n = 8), Carcass (n = 2), and VitE2 (n = 12). Based on fertility data, animals were grouped as Success (at least one pairing resulted in live birth), Fail (no live birth), and Unknown (animal not paired, Table 3). Differential expression analysis was then performed on these three diet groups using diet and fertility outcome as factors in a multi-factor design. No apparent grouping by fertility was observed after one-way hierarchical clustering analysis (Fig. 3a); however, 156 genes were identified as differentially expressed in Success vs Fail, with 91 being up- and 65 downregulated (Fig. 3b, Supplementary Table 3.3). Upregulated genes were enriched in terms related to translation initiation, integrin binding, and ubiquitination (Fig. 3c, Supplementary Fig. S6a); they formed predicted protein–protein interaction clusters enriched in translation initiation, focal adhesion and signaling (Fig. 3d, Supplementary Fig. S6b). Downregulated genes were enriched in calcium channel, RNA polymerase, autophagy and general terms related to transmembrane (Fig. 3c, Supplementary Fig. S6a); they formed predicted protein–protein interaction clusters enriched in the same terms except for general transmembrane (Fig. 3d, Supplementary Fig. S6b). Supplementary Archive S2 provides an interactive view of the networks and data tables. Overall, successful fertility outcome showed a strong association with semen transcriptome profiles related to translation initiation and focal adhesion.

**Table 3:** Information on fertility outcome for animals used in multi-factor differential expression (DE) analysis.

Study ID	Diet	Breeding history (study year)	Did not whelp, #	Whelped, #	Born, total	Fertility outcome (for DE analysis)
Car1	Carcass	Paired × 2	0	2	12	Success
Car2	Carcass	Paired × 3	0	3	10	Success
C01	Control	Paired × 2	0	0	0	Unknown*
C02	Control	Paired × 2	1	1	2	Success
C03	Control	Paired × 2	0	2	6	Success
C04	Control	Paired × 5	5	0	0	Fail
C06	Control	Paired × 8	1	7	33	Success
C07	Control	Paired × 4	2	2	8	Success
C08	Control	Paired × 5	1	4	11	Success
C10	Control	Paired × 2	2	0	0	Fail
VE2.01	VitE2	Paired × 4	4	0	0	Fail
VE2.02	VitE2	Not Paired	NA	NA	NA	Unknown
VE2.03	VitE2	Not Paired	NA	NA	NA	Unknown
VE2.04	VitE2	Paired × 2	0	2	8	Success
VE2.05	VitE2	Not Paired	NA	NA	NA	Unknown
VE2.06	VitE2	Not Paired	NA	NA	NA	Unknown
VE2.07	VitE2	Not Paired	NA	NA	NA	Unknown
VE2.08	VitE2	Paired × 4	4	0	0	Fail
VE2.09	VitE2	Paired × 7	2	5	8	Success
VE2.11	VitE2	Paired × 4	3	1	2	Success
VE2.12	VitE2	Paired × 3	2	1	6	Success
VE2.13	VitE2	Paired × 2	1	1	4	Success

*females did not ovulate; Paired x*—paired this amount of times with different females;

‘Whelped’ column indicates how many females gave birth after pairing with the corresponding male;

‘Did not whelp’ column indicates how many females did not conceive/not give birth after pairing with the corresponding male.

#### Effect of diet on semen transcriptome is stronger when factoring in fertility outcome

**Figure 4:**
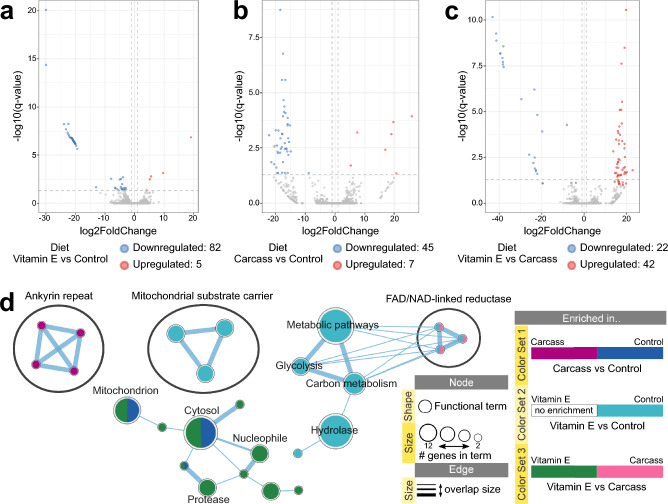
Linked effect of diet and fertility outcome in semen transcriptomes of black-footed ferrets. Volcano plot showing differentially expressed genes in comparison pairs (**a**) VitE2 versus Control, (**b**) Carcass versus Control and (**c**) VitE2 versus Carcass diets (Multi-factor design with Fertility and Diet, Wald test, q-value < 0.05, absolute fold change ≥ 2). (**d**) EnrichmentMap displays of enriched gene-sets based on differentially expressed genes between various diet comparison pairs. Supplementary Archive 7 contains the web session of the EnrichmentMap networks for interactive viewing.

The same animals and groupings were then used to compare the effect of diet on semen transcriptome with and without the fertility outcome factor. When only the diet was used in the statistical design, 19 genes were differentially expressed in VitE2 vs Control, 2 in Carcass vs Control, and only 1 in VitE2 vs Carcass (Supplementary Table S3.4). Multi-factor analysis including both diet and fertility outcome in the statistical design returned 87 differentially expressed genes in VitE2 vs Control (Fig. 4a), 52 in Carcass vs Control (Fig. 4b), and 64 in VitE2 vs Carcass (Fig. 4c). The majority of genes were enriched in the Control group and were related to mitochondria function and metabolism, including both carbon metabolism and glycolysis (Fig. 4d, Supplementary Fig. S7). Genes upregulated in VitE2 vs Carcass were also enriched in mitochondrion, as well as proteasome function (Fig. 4d). Supplementary Archive S2 provides an interactive view of the networks and data tables. Overall, diet had the most pronounced effect on semen transcriptome profiles when associated with fertility outcome and mainly affects terms related to mitochondrial function.

## Discussion

Here for the first time, we generated and analyzed the proteome and transcriptome of the ejaculate of the endangered black-footed ferret. Semen obtained from wild ferrets showed higher proteome variation with a predicted interaction cluster of ribosome proteins, which was absent in ex situ ferrets. Additionally, the semen transcriptome of wild ferrets was enriched in functions related to ribosomal RNA processing and potassium ion transport when compared against all ferret groups. Successful fertility outcome documented for ferrets in this study showed a strong association with genes enriched in translation initiation and focal adhesion. By itself, diet had a weak association with the semen transcriptome; however, introducing an additional factor of fertility into the analysis uncovered many genes related to mitochondrial function that seem to be influenced by the diet.

Semen has a very heterogeneous composition with a mixture of cellular (mainly spermatozoa, 5% of semen volume) and non-cellular (seminal fluid with secretions and extracellular vesicles from different accessory sex glands, 95% of semen volume) fractions^18^. Combining this with the fact of spermatozoa being the most diverse cell type known, reflecting species-specific environments and reproduction^28^, and the sperm transcriptome showing high species specificity except for some key similarities related to fertility^29^, the study of a species’ semen proteome and transcriptome becomes essential for understanding reproduction dynamics in the population. In our study of the black-footed ferret semen proteome, we identified 1757 proteins, out of which 1281 were previously reported in sperm^24,25^, 612 in seminal plasma^26,27^, and 590 reported in both. We also could identify genital tract markers for testis, epididymis and the prostate^26^, further showing the heterogeneity of the ferret ejaculate.

Proteins that we identified in the ferret ejaculate formed a predicted protein–protein interaction “hairball” network that could be further disassembled into clusters with various enriched terms and pathways integral to semen function and the fertilization process. The largest cluster with the most proteins and predicted interactions was enriched in mitochondrion function, including oxidative phosphorylation and glycolysis, while the next second largest cluster was enriched in proteasome and chaperon complexes. Various smaller clusters were related to sperm motility and capacitation, actin cytoskeleton, sperm-egg recognition and fusion, as well as RNA splicing and translation. All these clusters nicely represent the two distinct subcellular compartments of the sperm, the head and the tail. The sperm head consists of the nucleus with the paternal genome and the acrosome with hydrolytic enzymes for oocyte penetration. Proteins localized exclusively in the human sperm head showed enrichment in DNA packaging, RNA metabolism and nucleocytoplasmic transport^30^. The sperm tail consists of a flagellum involved in sperm motility and numerous mitochondria in the midpiece involved in energy production. In human sperm, proteins found exclusively in the tail show strong enrichment in oxidative phosphorylation pathways of mitochondria, as well as other energy generation processes such as the citrate cycle and fatty acid metabolism^31^. The proteasome complex has been shown to be localized in both sperm head and tail and to play crucial roles in sperm biogenesis during spermatogenesis^32^, as well as in processes of capacitation, acrosome reaction, egg-sperm interaction and protein processing after fertilization^33,34^. Finally, some predicted protein–protein interaction clusters in ferret semen showed enrichment in extracellular matrix processes, including cell adhesion, coagulation, extracellular transport, and defense response. In human semen, proteins from extracellular vesicles showed enrichment in functions important for vesicle transport, as well as in complement and coagulation pathways^35^, while proteins from seminal fluid in general are highly enriched in calcium ion binding, defense response and peptidase activity required for sperm motility and capacitation^18^.

Sperm has been shown to contribute not only DNA but a set of diverse RNAs to the oocyte upon fertilization ^36,37^ which mediate epigenetic inheritance^38–42^. For example, environmental effects of diet experienced by adult males in humans and other mammal species were shown to be passed to the next generation via small non-coding (snc) RNAs in sperm^41,43^. A recent study has revealed that the RNA profile in sperm includes not only sncRNAs and fragmented species of long RNAs, as was previously thought^44^, but 3440 intact transcript species of messenger (m) RNA and long non-coding (lnc) RNAs from 1624 genes in mouse and 4100 intact mRNAs and lncRNAs from 2205 genes in human sperm^45^. This profile of intact long RNAs is conserved in mice and humans, distinct from the testicular profile, and was shown to be functionally enriched for translation machinery^45^, suggesting that these RNAs are not simply leftovers from spermatogenesis^46^. Sun and colleagues propose that these intact RNAs enriched in translation are likely nonfunctional until after fertilization, where they meet the oocyte ribosomes and may contribute to quicker and more robust protein synthesis^45,47^, as well as alter the translation in early embryos^48^. Moreover, in a study on the ejaculate sperm from fertile men, 25 functional groups were identified, with the second most dominant group enriched in protein synthesis process^49^. Interestingly, in both our proteomic and transcriptomic results, semen from wild ferrets showed a clear distinction from ex situ animals by harboring more clusters of proteins and transcripts related to ribosomal machinery. We hypothesize that the sperm from wild ferrets delivers a more robust ribosomal machinery to the oocyte upon fertilization resulting in the better fertility outcome observed in the wild compared to ex situ ferrets (T. M. Livieri, Pers. comm.).

Semen from wild ferrets also showed enrichment in potassium ion channels compared to ex situ ferrets. Ion channels have an important role in sperm capacitation, affecting the acrosome reaction, sperm motility and other fertilization processes via regulation of sperm membrane potential, intracellular pH and Ca^2+^ levels^50,51^. Among them, potassium channels are crucial for fertility^52^ and range in type, with intracellular alkalinization-activated channels reported in sperm of mice^53^ and humans^54^, and voltage-gated ion channels recently reported in human sperm^55^. Here, in the semen of the wild ferrets we observed the enrichment of *KCNK12*, *KCNS1* and *KCNB2* genes which are members of the voltage-gated potassium ion channel family. A member of this family (*KCNQ1*) was reported to play a vital role during human sperm capacitation^55^, which leads us to hypothesize that another reason for improved fertility in wild compared to ex situ ferrets may lay in a more efficient sperm capacitation and motility hyperactivation upon entering the female genital tract.

A number of studies have shown an association of sperm RNA profile with fertility status in males^49,56,57^. In our study, we only had information on breeding success for captive males and, therefore, could not fully compare wild and ex situ ferrets in this metric. However, already among ex situ ferrets, fertility success showed a strong association with the semen transcriptome enriched in various processes related to sperm biogenesis and function. Interestingly, one such enriched process included translation initiation, which may either represent a piece of translational machinery being delivered to the oocyte after fertilization as discussed above for the wild ferrets, or the involvement in the sperm’s own protein synthesis process via the mitochondrial-type ribosomes that starts upon sperm capacitation in the female reproductive tract and contributes to fertilization success^58^.

It is important to note that in our study the diet by itself showed a weak association with the semen transcriptome in ex situ ferrets, however, this association became stronger after factoring in the fertility outcome for each male analyzed. There was no clear functional enrichment for a particular diet group, but our data analysis still showed that the mitochondrial metabolic function seems to be the one affected by the different diets. As mitochondrial production of energy in the sperm tail is tightly connected to sperm motility^59^, this observation may be related to the previously reported improved sperm motility index in ferrets kept on the beef and control with prey items (rat and hamster) diet compared to the control diet alone^10^.

One of the limitations of our study was the low availability of samples, which resulted in small volumes of ejaculate that could be collected for each analysis. For the proteomic analyses, we had to pool all samples for each diet group, therefore preventing us from performing quantitative analyses. At the same time, for the transcriptomic analysis, we were able to obtain enough semen to perform RNA-seq, but not enough to separate the ejaculate into spermatozoa and seminal fluid fractions. This led to an uneven sperm to seminal fluid ratio across the samples resulting in a high variation observed in our transcriptome data. In the future, obtaining more semen samples from more ferrets will allow us to examine sperm and seminal fluid separately and compare their proteome and transcriptome profiles quantitively between experimental groups. In addition, generating transcriptomes from reproductive tissues of the ferret will help determine which genes in the semen are remnants of spermatogenesis or epididymal sperm maturation, and which might be important during the transition of sperm in the female genital tract, fertilization and eventually embryo development.

As has been demonstrated in numerous studies, the “sperm RNA code” is sensitive to environmental factors and can impact the offspring’s phenotype^60^. Our observations on the semen proteome and transcriptome of black-footed ferrets suggest a similar importance of environmental regulation of sperm proteins and RNAs in fertility outcome, as well as a potential effect on the offspring phenotype, which would drive the reproductive fitness of wild ferrets further away from the ex situ population. These results add additional levels to the complicated phenomenon of environmental-dependent inbreeding depression^8^ which we hypothesize is happening in the ferret population and is affecting its fertility success. In the future, adding more generations of animals fed on various diets and focusing on additional species of RNAs as well as other types of omics data, such as metabolomics and epigenomics, while relating it all back to genomics would expand our understanding of the reproductive changes happening in the black-footed ferret population and help improve our conservation efforts for this unique endangered species.

## Materials and methods

### Ethical statement

This study was approved by the Lincoln Park Zoo Research Committee (Chicago, IL, proposal #2007–005). All trapping was authorized by the U.S. Fish and Wildlife Service (USFWS, Carr, CO) under permit #TE064682-1 and was conducted by personnel of the U.S. Forest Service, National Park Service and Prairie Wildlife Research as part of routine population monitoring. All animal experiments conformed to the Guide for Care and Use of Laboratory Animals and were approved by the Lincoln Park Zoo Research Committee and USFWS.

### Animals

Black-footed ferrets in this study were mature, breeding age males of various ages that were either caught in the wild (RMA, Rocky Mountain Arsenal, Colorado) or were part of an ex situ colony at two of the five breeding centers for the species (FCC, USFWS National Black-Footed Ferret Conservation Center, Carr, CO; LZG, Louisville Zoological Gardens, Louisville, KY, Table 1). The prime breeding age for males are ages two and three years old^61^.

Trapping and immobilization of wild ferrets followed protocols of the Black-Footed Ferret Recovery Implementation Team^62^ and Kreeger et al.^63^. Briefly, animals were cage-trapped at night and returned to the same location following examination and recovery from anesthesia, usually within 1 h of capture.

### Dietary treatments

Wild individuals (n = 4) were considered to be on a Wild diet, which typically consists of prairie dogs. Captive individuals were subjected to different dietary treatments (Table 1). The Control diet (n = 11) consisted of horsemeat with the addition of a prey item (rat or hamster) twice per week. The Vitamin E diet (n = 15) consisted of the Control diet supplemented with vitamin E (D-α-tocopherol; 400 IU/kg dry matter basis). Two of the ferrets on the Vitamin E diet were considered first generation (VitE1), as they switched to this diet from the Control diet during their lifetime. Thirteen ferrets were considered second generation (VitE2), i.e., they started the Vitamin E diet at birth and at least one of their parents was on the Vitamin E diet. The Carcass diet consisted exclusively of rat or mice carcasses, which was meant to emulate the Wild diet. Two ferrets did not have a consistent dietary treatment and were therefore labeled as Various.

### Semen collection and sperm evaluation

Testes firmness was used as an indicator of sperm production and breeding readiness in males^61^ and testis tumescence was checked via palpation for each ferret to decide the time of semen collection^64,65^. Semen was collected from wild and ex situ ferrets in March 2020 and April 2021, respectively. Males were subjected to anesthesia (Ketaject 38.8 mg/kg; diazepam 0.06 mg/kg) and semen was collected following existing protocols^66^. A rectal probe delivered electro-stimulations over a range of 2–5 V and semen was collected using a micropipette^66,67^.

After semen collection, cells were evaluated for percent motility, motility status (forward progression of 0–5; 5 is the highest score), normal morphology and concentration. Acrosomal integrity was assessed using phase-contrast microscopy (1000 ×) on 100 sperm cells per sample, as described previously^64,68^.

A small volume from each collected semen sample was processed and stored for proteomic and/or transcriptome analyses. For proteomic analysis, samples were spun down at 1000xG for 5 min (Eppendorf 5424 Microcentrifuge) and then resuspended in 50uL RIPA Lysis and Extraction Buffer (Thermo Fisher Scientific) supplemented with 1 × Halt™ Protease and Phosphatase Inhibitor Single-Use Cocktail (Thermo Fisher Scientific). After lysis overnight, samples were flash-frozen in liquid nitrogen. For transcriptome analysis, 3–5 μl of semen was collected using a micropipette after electroejaculation and directly added to vials of RNA-later solution (Invitrogen). Samples were allowed to equilibrate at room temperature for 5 min and then directly frozen in liquid nitrogen or placed on dry ice for future use.

### Protein digestion

Semen samples for proteomic analysis were collected from the animals listed in Table 1 and Supplementary Table S1. Samples were sent to the Proteomics Core Facility at the University of California, Davis Genome Center (https://proteomics.ucdavis.edu/) for processing and mass spectrometry. Because semen volume and protein amount in each sample were very small, samples from animals of the same diet group were pooled as shown in Supplementary Table S1.1. Proteins were subjected to tryptic digestion via suspension-trap (S-Trap) devices (ProtiFi, Fairport, NY). The obtained peptides were submitted to liquid chromatography-mass spectrometry (LCMS) analysis. Specifically, peptides were separated on a Thermo Fisher Scientific Dionex UltiMate 3000 RSLC system with a PepMap 75umx25cm C18 analytical column (PepMap, Denmark) preceded by a PepSep C18 guard column, all heated to 40 °C. A volume of 5 μl was injected, corresponding to 0.6 μg of total peptide, and separation was performed in a total run time of 60 min with a flow rate of 600 μl/min with mobile phases A: water/0.1% formic acid and B: 80%ACN/0.1% formic acid. Separated peptides were eluted directly into an Orbitrap Exploris 480 instrument (Thermo Fisher Scientific, Bremen, Germany). The full mass spectrometry (MS) resolution was set to 60,000 at m/z 200 and the full MS AGC target was 300% with an IT set to Auto. Mass range was set to 350–1500. For fragmentation spectra, at a resolution of 15,000, isolation width was set at 1.6 m/z and normalized collision energy was set at 30%. The AGC target value was set to Standard, with a maximum injection time of 40 ms and we did TopN = 30 scans.

### Proteome data processing and prediction of posttranslational modifications

Mass spectrometry raw files were processed with MSFragger (v. 18). For all searches, a protein sequence database for *Mustela putorius furo* (GCF_011764305.1) supplemented with predicted protein sequences for Protamine 1 and Protamine 2 of *Mustela nigripes* (kindly provided by Dr Sergei Kliver), as well as decoys and 115 common contaminant sequences, were used. Decoy sequences were generated and appended to the original database for MSFragger. A maximum of two missing cleavages were allowed, the required minimum peptide sequence length was 7 amino acids, and the peptide mass was between 500 and 5000 Da. Carbamidomethylation of cysteine residues was set as a fixed modification, and methionine oxidation and acetylation of protein N termini along with S, T, Y phosphorylation, were set as variable modifications. The initial maximum mass tolerances were 20 ppm for both the precursor and fragment ions. A reversed sequence library was generated and used to control the false discovery rate at less than 1% for peptide spectrum matches and protein group identifications. Decoy database hits, proteins identified as potential contaminants, and proteins identified exclusively by one site modification were excluded from further analysis. Label-free protein quantification was performed with the IonQuant algorithm (v. 8.9). All other MSFragger parameters were kept at their default values.

Generated proteomic data is publicly available via PRIDE (Accession: PXD044152). The output of the post-translation modifications analysis can be found in Supplementary Table S2.

### In silico protein–protein interaction analysis using STRING

In silico protein–protein interaction analysis was performed on the basis of the STRING database^69,70^ for the domestic ferret (*Mustela putorius furo*) unless specified otherwise. Interaction networks were built based on the list of identified proteins or selected genes (differential expression analysis) using StringApp (v. 2.0.0)^71^ in the Cytoscape software (v. 3.9.1)^72,73^ with the confidence cutoff score set to 0.4 unless specified differently. For large interconnected networks (hairballs), clustering was performed using the MCL cluster mode in the clusterMaker 2 app (v. 1.3.1)^74^ with the granularity parameter (inflation value) set to 2, array source set to stringdb::score, and the edge weight cutoff set to 0.4. Functional enrichment of formed clusters was performed using the domestic ferret genome as a background unless specified otherwise. Lists of enriched functional terms for each cluster were analysed with different levels of redundancy to identify representative terms.

### RNA isolation, library preparation and transcriptome sequencing

For RNA extraction, samples were thawed on ice. They were then washed with cold PBS and spun down in a centrifuge for 5 min at 1000 rpm to remove the buffer; this process was repeated 3 times. Cell lysis, cDNA conversion, fragmentation, and library preparation were done in single tubes using the QIAseq FX Single Cell RNA Library Kit (Qiagen). Final library amplification was performed using Q5 Hot Start polymerase (New England Biolabs), utilizing indexing primers and universal Illumina adapters obtained from the Functional Genomics Lab (FGL) at the University of California, Berkeley. Final libraries, which were constructed for each individual sample, were submitted to the FGL for library quality check via Bioanalyzer (Agilent) and subsequent 150 bp paired-end sequencing on Illumina NovaSeq 6000 S4 flow cells, attempting 37 million reads per library.

### Transcriptome data processing

Sequence data quality checking was conducted using FastQC (v. 0.11.9). Trimmomatic (v. 0.35.6)^75^ was used to remove adapter content, low quality reads, and artificial poly-G tails from all reads. Quality information for each sample after trimming is provided in Supplementary Table S3.1. The processed and filtered reads were then aligned to the chromosome-length genome assembly of *Mustela nigripes* (NCBI accession GCA_022355385.1) using STAR (v. 2.7.6)^76^. RSEM (v. 1.3.3)^77^ was used to refine STAR-mapped reads into transcript-level counts (transcripts per million, TPM). The code used for transcriptome data processing is available online at https://github.com/docmanny/BFF_Transcriptome_Analysis/.

### Differential expression analysis

Differential expression analysis was performed on transcriptome data obtained from 33 samples (Table 1) using the DESeq2 package^78^ in R (v. 4.2.1). Three samples were excluded from analysis due to low sperm concentration (C05, C09 and VE2.10). Genes with zero and low read counts were filtered out using the DESeq2 independent filtering step. For visualization, size factors were estimated from the count data and the Relative Log Expression (RLE) normalization was used to obtain regularized log-transformed values. These normalized values were then used for principal component analysis (plotPCA function in DESeq2 R package) and creation of clustered heatmaps (pheatmap R package). Likelihood-ratio tests (Diet effect, Wild group contribution) or Wald tests (Diet and Fertility association) were used on genes that passed an independent filtering step and resulting *p*-values were adjusted for multiple testing using the Benjamini–Hochberg procedure^79^. For likelihood-ratio testing, all diet groups were used with the Wild group set as reference: Wild (n = 4), Control (n = 8), Carcass (n = 2), VitE1 (n = 2), VitE2 (n = 12), Various (n = 2). Genes with adjusted *p*-values (q-value) < 0.05 were considered differentially expressed. For the Wald test, only diet groups with known fertility outcomes were used, including Control (n = 8), Carcass (n = 2) and VitE2 (n = 12, Table 3). For multi-factor analysis, the design formula in DESeq2 object included interaction terms of fertility outcome and diet; genes with a q-value < 0.05 and absolute fold change ≥ 2 were considered differentially expressed in each comparison pair.

### Functional enrichment analysis using DAVID and EnrichmentMap

Selected genes from differential expression analyses were used for gene-set functional enrichment analysis with the DAVID tool^80^, setting species to either domestic ferret or human. For each comparison pair, the total number of genes and separately up- and downregulated genes were analyzed. The EASE score (modified Fisher Exact p-value of enrichment) was set to 0.1. Functional enrichment networks were built based on DAVID output charts of gene-set enrichment for each comparison pair using the EnrichmentMap app^81^ (v. 3.3.4) in Cytoscape with the Overlap Coefficient set to 0.5 (domestic ferret background) or the Jaccard/Overlap Coefficient set to 0.375 (human background). The Autoannotate app (v. 1.3.5) with the MCL based on similarity coefficient was used to create annotated groups.

### Statistical analysis

Statistical analysis of transcriptome data is provided above. For sperm characteristics, data was visualized using the ggboxplot function from the ggpubr (v. 0.50) R package. The Kruskal–Wallis rank sum test was used to determine changes in each sperm characteristic across the different diets. The Wilcoxon rank-sum test was used for *post-hoc* pairwise comparison of diet groups (p-value adjustment: Benjamini-Hochberg). Adjusted *p*-values < 0.05 were considered statistically significant.

### Supplementary Information

Supplementary Information 1.

Supplementary Information 2.

Supplementary Information 3.

Supplementary Information 4.

Supplementary Information 5.

Supplementary Information 6.

## Data Availability

All proteome and transcriptome data were generated in the current study and deposited to PRIDE (Accession: PXD044152) and NCBI SRA repository (BioProject PRJNA997940), respectively. The rest of the data generated and analyzed in this study is included in this published article and its Supplementary files: (1) data on identified proteins, predicted posttranslational modifications and differential expression analysis is available in Supplementary Tables S1-S3; (2) interactive view of networks with corresponding data tables is available in Supplementary Archives S1-S2.
