# Supplementary material for: Semen proteome and transcriptome of the endangered black-footed ferret (Mustela nigripes) show association with the environment and fertility outcome
Source: Sci Rep. 2024 Mar 25;14:7063. doi: 10.1038/s41598-024-57096-w (PMC10963785; doi:10.1038/s41598-024-57096-w)
Supplement: Supplementary file 1 — Supplementary Information 1. [file 41598_2024_57096_MOESM1_ESM.pdf]

Supplementary Figure S1

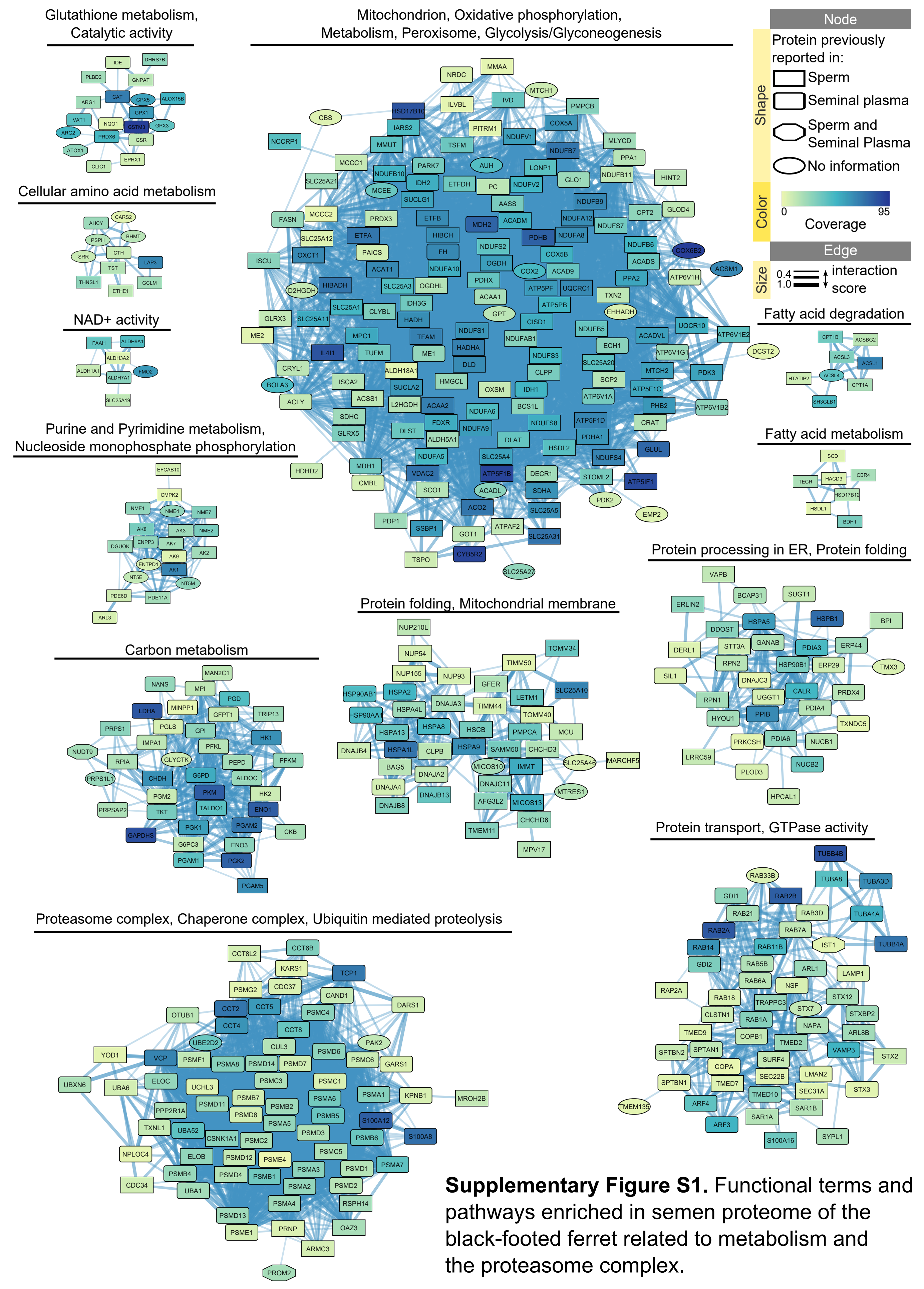

Supplementary Figure S1. Functional terms and pathways enriched in semen proteome of the black-footed ferret related to metabolism and the proteasome complex.

Supplementary Figure S2

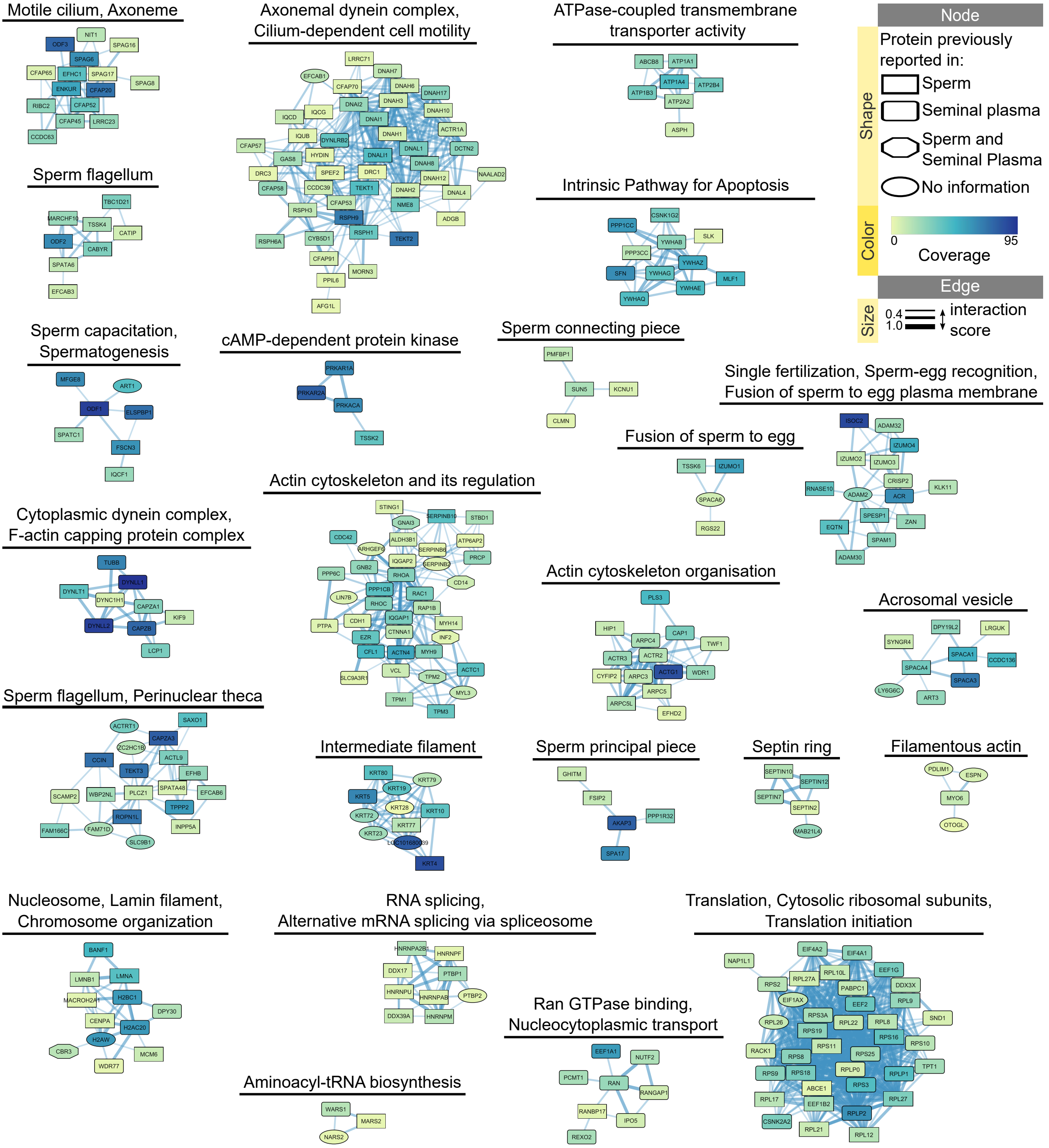

Supplementary Figure S2. Functional terms and pathways enriched in semen proteome of the black-footed ferret related to sperm structure, motility, capacitation, fertilization and translation.

Supplementary Figure S3

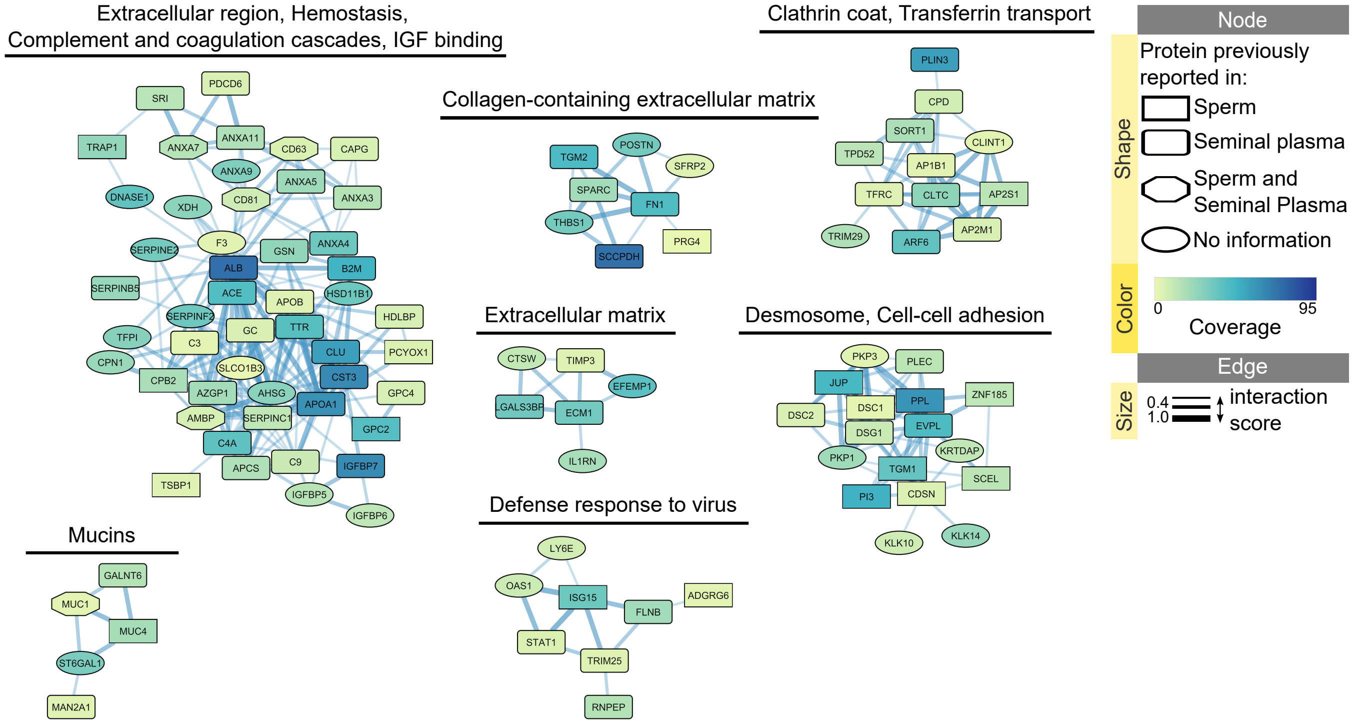

Supplementary Figure S3. Functional terms and pathways enriched in semen proteome of the black-footed ferret related to extracellular space.

Supplementary Figure S4

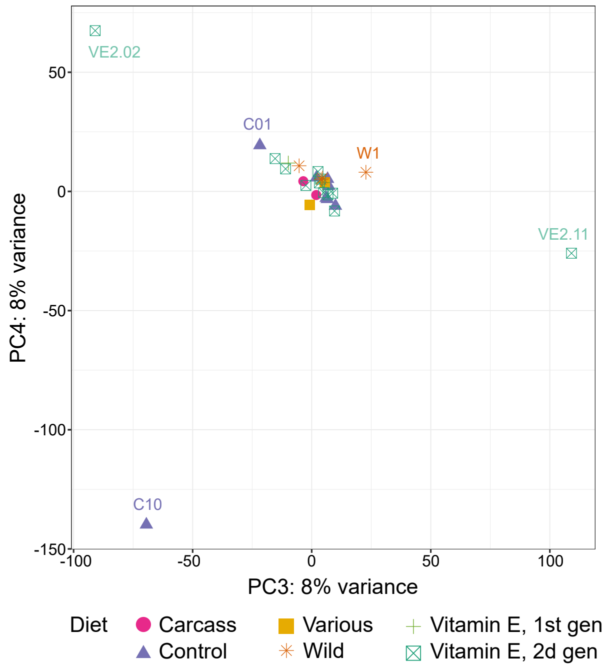

Supplementary Figure S4. Principal components 3 and 4 representing variation in semen transcriptome of all diet groups.

Supplementary Figure S5

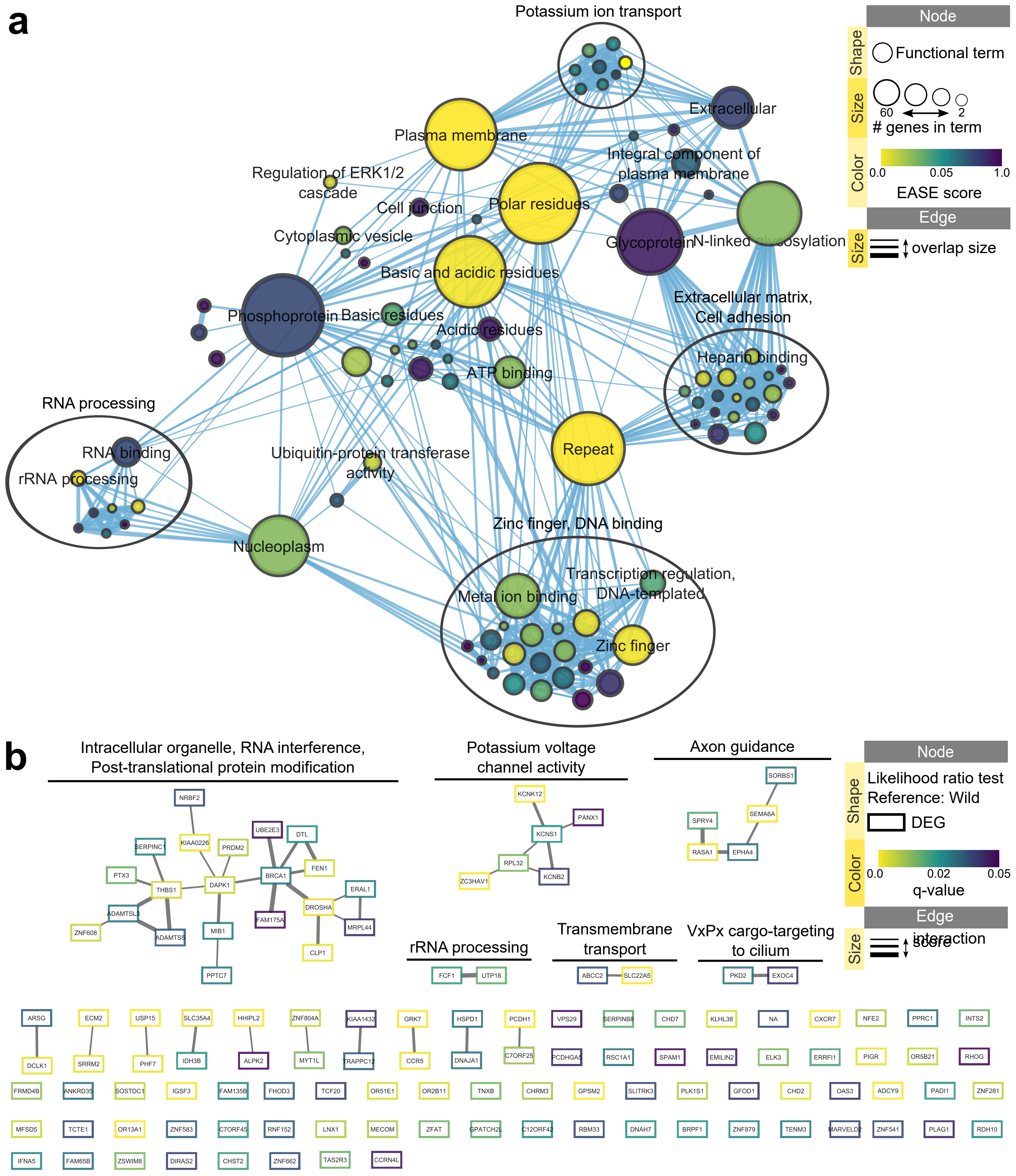

Supplementary Figure S5. (**a**) EnrichmentMap displaying enriched gene-sets and (**b**) STRING network of predicted protein interactions based on differentially expressed genes between full (all diet groups) and reduced (Wild excluded) model.
Background for enrichment is set to *homo sapiens*.

Supplementary Figure S6

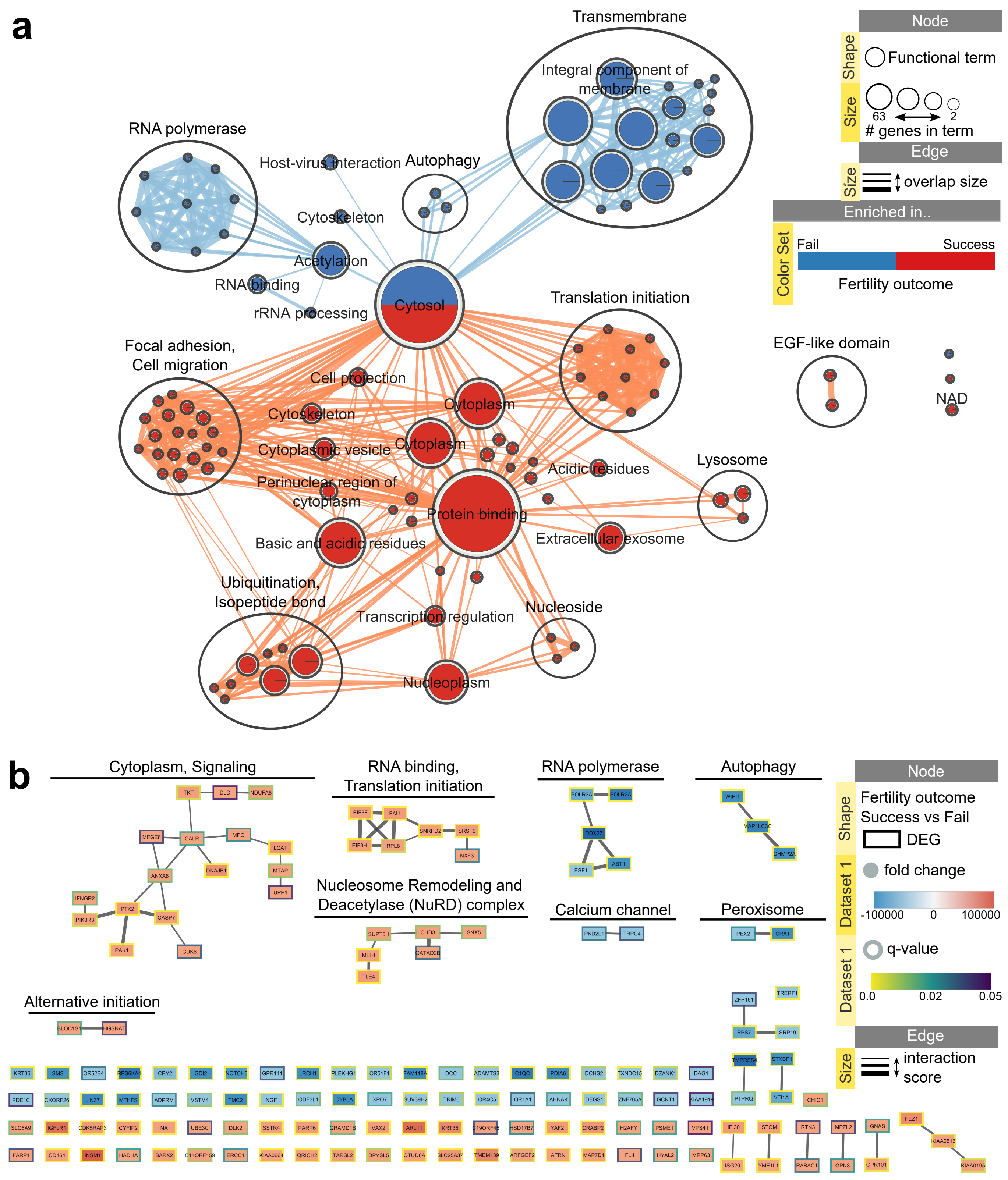

Supplementary Figure S6. (**a**) EnrichmentMap displaying enriched gene-sets and (**b**) STRING network of predicted protein interactions based on differentially expressed genes in semen of ferrets with success vs fail fertility outcome.
Background for enrichment is set to *homo sapiens*.

Supplementary Figure S7

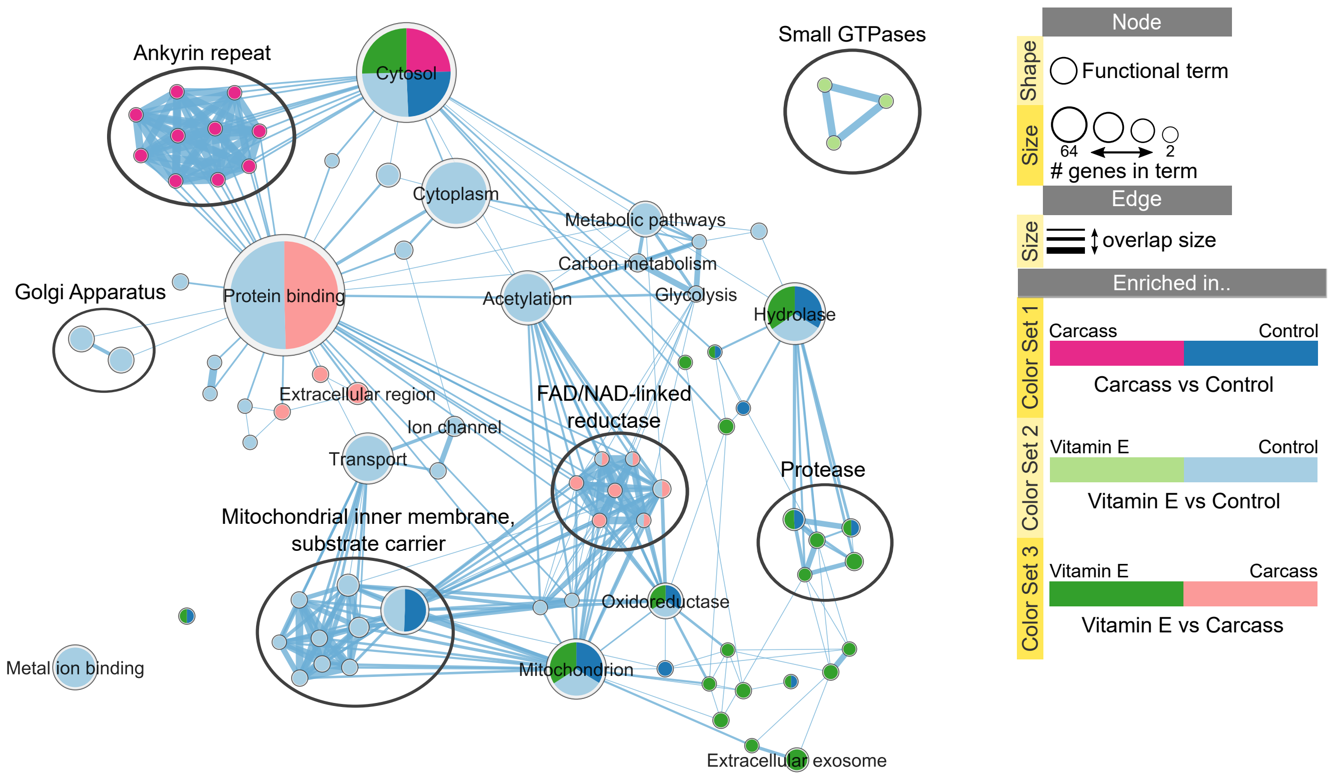

Supplementary Figure S7. EnrichmentMap displays enriched gene-sets based on differentially expressed genes between various diet comparison pairs with relation to fertility success. Background for enrichment is set to *homo sapiens*.
